# The Impact of Post-Operative Radiotherapy in Early Stage (pT1-pT2N0M0) Oral Tongue Squamous Cell Carcinoma in Era of DOI

**DOI:** 10.3390/cancers13194851

**Published:** 2021-09-28

**Authors:** Daniela Alterio, Pasqualina D’Urso, Stefania Volpe, Marta Tagliabue, Rita De Berardinis, Matteo Augugliaro, Sara Gandini, Fausto Antonio Maffini, Roberto Bruschini, Irene Turturici, Stefano Riccio, Luca Calabrese, Alessia Farneti, Anna Starzyńska, Annamaria Ferrari, Barbara Alicja Jereczek-Fossa, Mohssen Ansarin, Giuseppe Sanguineti

**Affiliations:** 1 Division of Radiation Oncology, IEO European Institute of Oncology, IRCCS, 20141 Milan, Italy; daniela.alterio@ieo.it (D.A.); matteo.augugliaro@ieo.it (M.A.); irene.turturici@ieo.it (I.T.); annamaria.ferrari@ieo.it (A.F.); barbara.jereczek@ieo.it (B.A.J.-F.); 2 Department of Radiation Oncology, IRCCS Regina Elena National Cancer Institute, 00144 Rome, Italy; pasqualina.durso@ifo.gov.it (P.D.); alessia.farneti@ifo.gov.it (A.F.); giuseppe.sanguineti@ifo.gov.it (G.S.); 3 Department of Oncology and Hemato-Oncology, University of Milan, 20122 Milan, Italy; 4 Division of Otolaryngology and Head and Neck Surgery, European Institute of Oncology IRCCS, 20141 Milan, Italy; rita.deberardinis@ieo.it (R.D.B.); roberto.bruschini@ieo.it (R.B.); stefano.riccio@ieo.it (S.R.); mohssen.ansarin@ieo.it (M.A.); 5 Department of Biomedical Sciences, University of Sassari, 07100 Sassari, Italy; 6 Department of Experimental Oncology, IEO European Institute of Oncology, IRCCS, 20141 Milan, Italy; sara.gandini@ieo.it; 7 Division of Pathology, IEO European Institute of Oncology, IRCCS, 20141 Milan, Italy; fausto.maffini@ieo.it; 8 Division of Otorhinolaryngology, San Maurizio Hospital, 39100 Bolzano, Italy; dott.lucacalabrese@gmail.com; 9 Department of Oral Surgery, Medical University of Gdańsk, 80-211 Gdańsk, Poland; anna.starzynska@gumed.edu.pl

**Keywords:** oral tongue cancer, post-operative radiotherapy, depth of infiltration, perineural invasion

## Abstract

**Simple Summary:**

The aim of the present study was to clarify the role of depth of infiltration (DOI) as an independent prognosticator in early stage (T1-T2N0M0) oral cavity tumors. Moreover, whether patients upstaged to pT3 for DOI > 10 mm need postoperative radiotherapy (PORT) in the absence of other risk factors has not been established yet. The DOI alone was not sufficient to impact the prognosis and therefore other risk factors should be considered to indicate PORT indications in upstaged patients due to DOI > 10 mm.

**Abstract:**

*Background:* This study investigated the role of depth of infiltration (DOI) as an independent prognosticator in early stage (T1-T2N0M0) oral cavity tumors and to evaluate the need of postoperative radiotherapy in the case of patients upstaged to pT3 for DOI > 10 mm in the absence of other risk factors. *Methods*: We performed a retrospective analysis on patients treated with surgery and re-staged according to the 8th edition of malignant tumors classification (TNM). The role of DOI as well as other clinical/pathological features was investigated at both univariable and multivariable analyses on overall survival (OS), disease free survival (DFS), relapse free survival (RFS), and local RFS. *Results*: Among the 94 included patients, 23 would have been upstaged to pT3 based on DOI. Multivariable analysis showed that DOI was not an independent prognostic factor for any of the considered outcomes. The presence of perineural invasion was associated with a significant worse RFS (*p* = 0.02) and LRFS (*p* = 0.04). PORT was found to be significantly associated with DFS (*p* = 0.04) and RFS (*p* = 0.06). *Conclusions*: The increasing DOI alone was not sufficient to impact the prognosis, and therefore, should not be sufficient to dictate PORT indications in early-stage patients upstaged on the sole basis of DOI.

## 1. Introduction

Radical-intent surgery represents the standard treatment for oral tongue squamous cell carcinomas (OTSCCs) [1]. Post-operative radiotherapy (PORT) is indicated in the presence of risk factors such as advanced tumor stage (pT3, pT4) and nodal involvement (pN > 1, extracapsular extension). Moreover, for small (pT < 4 cm) and node-negative tumors, further prognosticators encompass both the status of surgical margins and histopathological features including perineural–lympho–vascular invasions (PNI and LVI, respectively) and high grade [1].

The last edition of the TNM by the American Joint Committee on Cancer (AJCC, 8th edition) introduced the depth of infiltration (DOI) as a novel prognostic factor for oral cavity cancers, thus leading to a redefinition of previous staging categories [2]. Specifically, small OCSCCs previously classified as pT1–pT2 (tumor < 4 cm) are currently upstaged to pT3 if DOI > 10 mm, with a clinically meaningful impact on the patients’ management and expected prognosis. Nevertheless, although DOI allowed us to improve discrimination among different stages, for small tumors, in the absence of any additional risk factors, DOI did not seem to improve the prediction of 5-year disease-specific mortality [3]. This finding raises the question on the independent prognostic role of DOI when compared to the other already known pathologic prognosticators such as PNI, LVI, surgical margins, and tumor grading in this subset of patients.

Moreover, the role of PORT in early-stage oral cavity tumors after radical surgery is still being debated. The main pathological factors considered to indicate an adjuvant treatment are PNI and close margins [4]. Regarding the role of DOI, a clear cut-off value of tumor infiltration identifying patients that may benefit from PORT has not been identified yet. Therefore, whether upstaged pT3 patients in the 8th TNM edition need PORT as a treatment intensification strategy is currently a matter of debate [3,5].

To investigate these controversial issues, we performed a retrospective analysis on patients with small OTSCC (maximum diameter < 4 cm) with negative lymph-node status aiming to evaluate the impact of DOI on patients’ outcome alone and compared to other known pathological risk factors and the impact of PORT in this population was also investigated.

## 2. Materials and Methods

We retrospectively reviewed all consecutive cases of early-stage OTSCC (AJCC 7th) treated at two Italian Institutions (European Institute of Oncology IRCSS-IEO in Milan and IRCCS Regina Elena National Cancer Institute in Rome) between 2014 and 2019. Indications to treatment, surgical technique, pathological analysis, and PORT were similar between the two Institutions according to international NCCN guidelines [1].

Only records from patients who had provided written informed consent to the anonymous use of clinical data for clinical research purposes were screened; the study received approval from the Ethical Committee (notification number: 225).

Only adult patients staged as pT1-pT2 (<4 cm) c/pN0 cM0 per the AJCC 7th and treated with radical surgery +/− PORT were considered eligible, provided they had a minimum follow-up of six months since the completion of primary treatment. Exclusion criteria were as follows: advanced-stage tumors (pT3, pT4), clinical or pathological nodal involvement, non-squamous histology, recurrent/second primary tumors, non-invasive disease, presence of synchronous metastases, and any previous local treatment in the head and neck region.

Clinical and pathological data were retrieved for electronic medical charts. At IEO Pathological specimens were reviewed by a dedicated pathologist with expertise in head and neck cancers (F.A.M.)., according to the published definition of DOI [6]. At Regina Elena National Cancer Institute, the DOI was already evaluated as a part of daily clinical practice. In OTSCC, DOI is defined as the distance between the level of the basement membrane of the closet adjacent normal mucosa and the deepest point of tumor invasion. Risk factors considered for PORT were close/positive surgical margins, PNI, LVI, and high tumor grading (G3).

Loco-regional staging procedures included both clinical examination and radiological imaging (magnetic resonance-MR- and/or computed tomography-CT- and/or neck ultrasonography-US).

### 2.1. Treatment Strategy and Follow Up

Treatment strategy was defined in the context of a multidisciplinary tumor board according to institutional guidelines. Radical-intent transoral surgery was performed in all cases. If positive surgical margins were detected at the definitive pathological specimen, re-resection was performed whenever possible; similarly, a selective prophylactic neck dissection was planned in case tumor infiltration > 3 mm.

PORT was indicated in the case of at least one of following prognostic factor (mainly close/surgical positive margins not suitable for a second surgical time, PNI, LVI). Moreover, patient’s characteristics and history as well as the applied surgical procedures were also considered to define indication for adjuvant treatments. When indicated, PORT was planned to begin within 6–8 weeks from the surgical procedure.

After treatments, all patients were followed up every three months for the first two years, every 4–6 months for the subsequent three years, and once a year thereafter. Clinical and radiological imaging (MR and/or CT and/or US) were required on a regular basis. At least a radiography and/or a CT of the chest was required once/year.

### 2.2. Statistical Analysis

Patient’s clinical–pathological and tumor characteristics were expressed as absolute and relative frequencies when variables are categorized or as median and interquartile range when presented as continuous variables. We used the Chi-square test and Wilcoxon rank test to investigate differences in prognostic factors by DOI value considering DOI both as the categorical and continuous variable. Relapse free survival (RFS) was defined as time form surgery util date of any relapse or last follow up. Local relapse free survival (LRFS) was defined as time from surgery until date of relapse on T or N or last follow-up. Disease free survival (DFS) was defined as time from surgery until date of any event or last follow-up. Overall survival (OS) was defined as time from surgery until date of death or last follow-up. Univariate models were performed to evaluate the association of demographic characteristics and other prognostic factors with clinical outcomes (DFS, RFS, LRFS, or OS). Differences between Kaplan–Meier survival curves were investigated with Log-rank tests. Multivariable Cox Proportional Hazard models were adopted to investigate the independent role of DOI adjusting for other significant prognostic factors. Hazard ratio (HR) with 95% confidence intervals (CIs) from multivariable Cox proportional hazard models were reported. All analyses were carried out with SAS and *p*-values < 0.05 were considered statistically significant.

## 3. Results

A total of 94 (78 from European Institute of Oncology and 16 from Regina Elena National Cancer Institute) patients with early stage OTCCs met the inclusion criteria and their data were analyzed. Patient, tumor-, and treatment-related characteristics are detailed in Table 1. 

The cohort showed an equal distribution per gender and a median age of 63 years. About 79% were smokers or former smokers. Considering treatment modalities, among the 43 patients treated with neck dissection, 18 (42%) received PORT. Considering the remaining 51 patients who did not undergo neck dissection, none of them received indication to PORT.

### 3.1. Postoperative Radiotherapy (PORT)

All 18 patients who had received PORT had a DOI > 10 mm, while among the 76 patients who did not receive PORT, only five (7%) had a DOI > 10 mm. The majority (78%) of patients submitted to PORT had at least one risk factor, while the majority (79%) of patients not submitted to PORT had no risk factors. Data on radiation treatment were available for 17 patients (one patient has been treated outside). All but one patient was treated with intensity modulated radiotherapy. The median time interval between surgery and the beginning of PORT was 88 days (range 57–123 days). The median total dose was 60 Gy (range 60–70 Gy), all administered with a standard fractionation schedule. The median overall treatment time was 42 days (range 39–51 days).

### 3.2. Stage Migration

Table 2 summarizes stage migration when DOI is considered (AJCC 8th edition). Overall, 31% and 51% of patients staged as pT1 and pT2 per AJCC 7th resulted in being upstaged to pT3 due to DOI, respectively. On the other hand, among the 23 patients staged as pT3 AJCC 8th, seven (30%) and 16 (70%) patients were ex-pT1 and ex-pT2, respectively.

### 3.3. Tumor-Related Risk Factors

**Figure 1:**
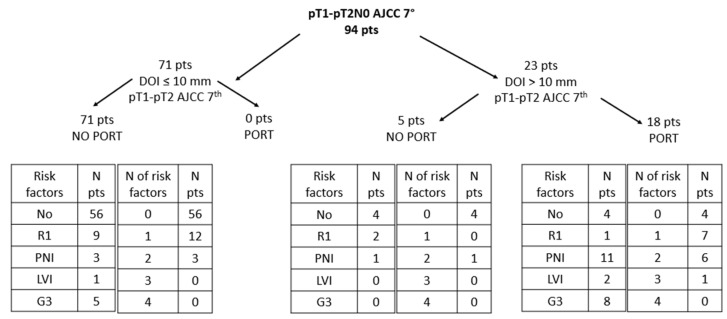
Distribution of pathologic risk factors according to depth of infiltration (DOI) and postoperative radiotherapy (PORT). AJCC = American Joint Committee on Cancer, G3 = grade 3, LVI = lymph vascular invasion, N = number, PNI = perineural invasion, pts = patients, R1 = positive/close surgical margins.

PNI resulted in being the most frequent risk factor, occurring in 16% of patients. Tumor grading was available for 86 patients with 13 of them having a G3 cancer. Overall, the majority of patients (68%) had none of the considered risk factors. Distribution of risk factors across the whole cohort is reported in Table 3. Among the 11 patients with close/positive margins (R1), eight patients were submitted to a second surgery, achieving a negative margin. One patient due to age and comorbidities and one patient with close (<5 mm) surgical margins were followed-up while one patient with close surgical margins was treated with PORT due to the concurrent presence of PNI. Distribution of pathological risk factors (close/positive surgical margins, perineural invasion, lympho-vascular invasion and grade 3) according to DOI and PORT has been reported in Figure 1. Among 14 patients with DOI < 10 mm and only one risk factor, half of them (7/14, 50%) had close/positive surgical margins. Among patients with DOI > 10 mm who were not treated with PORT, there was only one patient who had two risk factors (R1 and PNI), while among those treated with PORT and with one risk factor (seven patients), four of them had PNI, while five patients had both PNI and G3.

### 3.4. Association between DOI and Other Tumor-Related Risk Factors

The median value of DOI was 5 mm (IQR: 2–11). Tumors with DOI < 5 mm, between 5 and 10 mm, and >5 mm were 42 (44%), 26 (27%), and 27 (28%), respectively. Considered as the continuous variable, the increasing value of DOI was found to be strongly associated with PNI (with a median DOI of 4.25 mm for those with no PNI and of 12 mm for those with PNI, *p* < 0.0001, Wilcoxon rank test). When DOI was considered as a categorical variable (<5 mm, 5–10 mm, and >10 mm), a statistically significant association was maintained with the PNI (*p* < 0.0001, Chi-square test). Moreover, a significant correlation with high histological grade (G = 3) was identified (*p* = 0.0005). Conversely, no association was found between DOI and LVI (*p* = 0.22) as well as with the status of surgical margins (*p* = 0.27).

### 3.5. Clinical Outcomes

**Figure 2:**
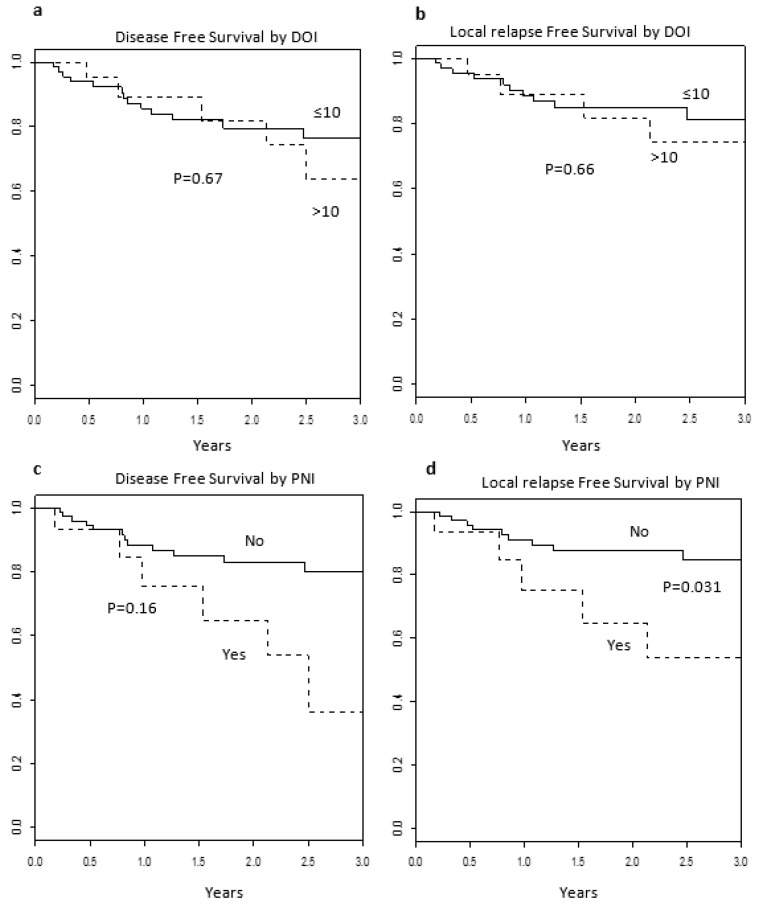
Disease free survival and local relapse free survival according to DOI (**a**,**b**) and PNI (**c**,**d**). PNI = perineural invasion, DOI = depth of infiltration.

Median follow-up was 24 months (range 0.5–68 months). At last follow up, 87 (92%) of patients were alive and free from disease. The 2y OS, DSS, and LRC were 92%, 75%, and 84%, respectively. Six (6%) and eight (8%) patients experienced a second primary tumor and lymph node recurrence, respectively. One patient had both second primary tumor and lymph node recurrence. Four patients developed distant metastases with also a loco-regional recurrence. Among the 51 patients who did not receive neck dissection, 12 (24%) patients experienced disease recurrence, which was local in three cases, regional in five, and distant in four. In contrast, of the 43 patients submitted to neck dissection, seven (16%) experienced any recurrence event (three local, three regional, and one loco-regional). Loco-regional recurrences were not statistically associated with neck dissection (*p* = 0.82). If we consider indication to PORT (presence of at least one risk factor), we obtained four subgroups: 59 patients with no indication who have been not treated with PORT; four patients with no indication who have been treated with PORT; 14 patients for whom PORT was indicated but have been not treated with PORT; and 17 patients who, albeit indicated, were not treated with PORT. Subgroup analysis did not show any significant correlation with the considered outcomes. DOI was found to not be associated with either OS (*p* = 0.45), DFS (*p* = 0.67) or LRFS (*p* = 0.66) (Figure 2a,b). Moreover, when DOI was categorized as <5 mm, 5–10 mm, and >10 mm (42, 26, and 26 patients, respectively), we did not find any significant difference in terms of both OS and DFS (Log-rank *p* = 0.945 and *p* = 0.515, respectively). Similarly, PNI was not found to be correlated with OS (*p* = 0.15) and LRFS (0.03), but was not found to be significantly associated with DFS (*p* = 0.16) (Figure 2c,d), even if patients with PNI had a significant greater probability of local relapse (*p* = 0.028). Of the 18 patients treated with PORT, those without PNI (*n* = 7) did not experience any local recurrence, while those with PNI (*n* = 11) had three events. All other risk factors (positive margins, LVI, and grading) and PORT were not found to be significantly associated with any of the considered oncological outcomes. Among the 24 patients with DOI > 10 mm, PORT was not associated with either overall survival or any progression free survival.

### 3.6. Multivariable Analysis

Results of the multivariable analysis were reported in Table 4. PNI was not included in the model since it was not found to be correlated with OS (long rank test *p* = 0.21). Multivariable Cox proportional model showed that DOI is not a significant independent prognostic factor for any of the considered outcomes. On the other hand, the absence of PNI resulted in being associated with 75% less probability of relapse (*p* = 0.02 for RFS and *p* = 0.04 for LRFS). All other risk factors (close/positive surgical margins, LVI, and tumor grading) were not independent prognostic factors for any of the considered outcomes. Similarly, neck dissection (performed or not) in this selected group of patients was not found to be an independent prognostic factor for locoregional recurrence (*p* = 0.85). The multivariate analysis showed that PORT represents an independent prognostic factor, with patients who were not treated with PORT experiencing a worse DFS (*p* = 0.04) and a trend to a worse RFS (*p* = 0.06).

## 4. Discussion

The results of our analysis demonstrate that only PNI is an independent prognostic factor in early stage (T < 4 cm and N0) OTSCCs, considering DOI along with other well-known pathological tumor-related risk factors. To the best of our knowledge, this is the first study suggesting that PNI can play a role as a stronger prognosticator than DOI in early stage OTSCCs. Correlation between DOI and patient prognosis has been reported by several authors [3,7,8,9,10,11,12,13,14]. Moreover, a recent meta-analysis confirmed that DOI was associated with a greater chance to develop nodal recurrences, and with a detrimental effect on survival in early-stage OCSCCs [14]. Nevertheless, whether DOI represents an independent prognostic factor in such a cohort of small tumors is still a matter of debate. In their retrospective analysis, Ebrahimi et al. reported that in a cohort of 1409 OTSCC patients, DOI seemed to be strongly correlated with other risk factors including primary tumor size, pN category, ECE, and close or positive surgical margins (*p* < 0.001 for all factors) [3]. Interestingly, authors performed a prior subset analysis on 769 low risk (negative lymph-node and surgical margins) patients with small (T < 4 cm) tumors, showing no statistically significant difference in 5-year disease-specific mortality among patients with DOI < 5 mm and those with DOI > 10 mm (6% vs. 10%, respectively, *p* = 0.169), in the absence of other risk factors. Therefore, the authors suggest that a worsening prognosis related to increasing DOI could be primarily due to other-than-DOI prognostic factors (3). Unfortunately, these factors were not available for that analysis and further studies on the topic are needed. In the current series, all risk tumor-related factors (DOI > 10 mm, surgical margins, PNI, LVI, and grading) were collected and analyzed. Our results showed that increasing DOI was not associated with a worsening in patient outcomes. This finding could be at least partially explained by the widespread distribution of risk factors between the two group of patients (DOI < 10 mm and >10 mm). On the other hand, our data confirmed a strong association of DOI with other risk factors such as PNI and tumor grading. Additionally, the multivariate analysis revealed a deterioration in patient prognosis associated with the presence of PNI and not to DOI, regardless on whether the latter was considered as a continuous or a categorical variable. Therefore, our work provides the theoretical basis to confirm that other-than-DOI factors (specifically PNI) must be considered for patient prognosis in early stage OTSCC. To explain the lack of association between DOI and patient outcome, some authors have hypothesized that DOI might mirror not only a more aggressive tumor biology, but also a delayed patient presentation at diagnosis [3]. Indeed, some patients with thicker primary tumors could have biologically indolent disease. In contrast, the presence of PNI might be a feature of tumor aggressiveness and therefore be a stronger determinant for patient prognosis. Indication to PORT for early stage (pT1–T2 N0 M0, AJCC 7th) OTSCC tumors is nowadays controversial [4]. Despite tumor infiltration being a well-known prognosticator in OTSCC a defined cut-off value for the identification of patients with high risk who may benefit from PORT has not been determined yet [14,15,16]. Cramer et al. analyzed the impact of PORT in 823 patients upstaged to pT3N0M0 AJCC 8th for DOI excluding concurrent LVI and positive margins [17]. Results showed that PORT was associated with an improved OS (adjusted hazard ratio [aHR] 0.47, 95% confidence interval [CI] 0.30–0.73). Nevertheless, Ebrahimi et al. showed that in the absence of risk factors (nodal involvement and positive surgical margins), the 5-year disease specific mortality in patients with DOI > 10 mm was only of 2%, regardless of the execution of PORT [3]. Considering the totality of our cohort of 94 patients, PORT resulted in being significantly associated with DSF as well as with a trend toward a better DSFS. Nevertheless, PORT failed to impact on oncological outcomes in the case of DOI > 10 mm. However, it must be noted that as no patient with DOI < 10 mm received PORT and due to the widespread presence of pathological risk factors among our patients, it was not possible to perform any subgroup analysis to further investigate the role of PORT in the case of specific risk factor combinations. Specifically, due to the limited number of patients submitted to PORT with or without PNI (one patient vs. 17 patients), our analysis did not allow us to draw any conclusions on the role of PORT according to the presence of PNI. 

Overall, our results suggest that increasing DOI alone was not sufficient to impact patient prognosis, and therefore it should not be sufficient to dictate PORT indications in early-stage patients upstaged to pT3 in the TNM 8th edition on the sole basis of DOI; therefore, we can conclude that other prognosticators (particularly PNI) should be considered to select the best adjuvant strategy. However, it is important to note that while OTSCC represents the most common cancers of the oral cavity, our results could not be directly translated to other subsites (e.g., gingiva, floor of mouth).

The major pitfalls of our study are its retrospective nature, lack of centralized pathology revision, and the lack of a pathological nodal staging for half of the patients. Moreover, other prognosticators such as the pattern of invasion, PNI density foci, and tumor budding, which were recently demonstrated to be expression of tumor biological behavior, were not available [18,19,20]. 

## 5. Conclusions

The increasing DOI alone was not sufficient to impact the prognosis, and therefore it should not be sufficient to dictate PORT indications in early-stage patients upstaged on the sole basis of DOI. Nevertheless, our study represents a comprehensive analysis of all prognosticators actually available in everyday clinical practice for early-stage tumors, providing a useful tool to help clinicians in the decision-making process for the management of early-stage tumors in the era of DOI.

## Figures and Tables

**Table 1:** Tumor and treatment characteristics.

Characteristics		Number of Patients *n* = 94 (100%)
Age	Median, quartile	63, 73–43
Gender, *n* (%)	Female	46 (49)
	Male	48 (51)
Tobacco smoking status, *n* (%)	Yes	20 (21%)
	No	42 (45%)
	Former	32 (34%)
Alcohol consumption, *n* (%)	Yes	21 (22%)
	No	72 (77%)
	Former	1 (1%)
TNM classification (AJCC 7th), *n* (%)	pT1N0M0	59(63%)
	pT2N0M0	35 (37%)
TNM classification (AJCC 8th), *n* (%)	pT1N0M0 (Stage I)	42 (45%)
	pT2N0M0 (Stage II)	29 (31%)
	pT3N0	23 (24%)
Neck Dissection, *n* (%)	Yes	43 (46%)
	No	51 (54%)
Adjuvant treatment, *n* (%)	No	76 (81%)
	PORT	17 (18%)
	PORT + Chemother	1 (1%)

AJCC = American Joint Committee on Cancer, TNM: Tumor, Node, Metastases, PORT: postoperative radiotherapy.

**Table 2:** Stage migration from AJCC 7th to AJCC 8th.

Heading		AJCC 8th Ed			
**AJCC 7th Ed**		T1 (100%)	T2 (100%)	T3 (100%)	Total
	T1	41 (98%)	11 (38%)	7 (30%)	59 (63%)
	T2	1 (2%)	18 (62%)	16 (70%)	35 (37%)
	Total	42 (45%)	29 (31%)	23 (24%)	94 (100%)

**Table 3:** Description of risk factors according to depth of infiltration (DOI), postoperative radiotherapy (PORT), and neck dissection.

Characteristics		Total (*n* = 94 (%))	ND (*n* = 42)	No ND (*n* = 52)
Risk Factor	R1	11 (12%)	3	8
	R0	83 (88%)	39	44
	PNI	15 (16%)	11	4
	No PNI	79 (84%)	31	48
	LVI	3 (3%)	3	0
	No LVI	91 (97%)	39	52
	G3	13 (14%)	8	5
	G1–G2	73 (78%)	33	40
	Unknown	8 (8%)	1	7
No. of Risk Factors	0	64 (68%)	26	38
	1	19 (20%)	8	11
	2	10 (11%)	7	3
	3	1 (1%)	1	0

R1: close/positive surgical margins, R0: free surgical margins, PNI: perineural invasion, LVI = lympho-vascular invasion, G3: Grade 3; G1–G2: Grade 1–2, ND: neck dissection.

**Table 4:** Multivariable Cox proportional hazard models.

			All	Events	HR	Low 95%CI	Up 95%CI	*p*-Value
DFS	DOI	Continuum	94	25	1.11	0.99	1.24	0.06
	PORT	No	76	21	1			
		Yes	18	4	0.20	0.05	0.92	0.04
	PNI	No	79	19	1			
		Yes	15	6	2.38	0.83	6.82	0.11
RFS	DOI	Continuum	94	19	1.08	0.94	1.23	0.26
	PORT	No	76	16	1			
		Yes	18	3	0.19	0.04	1.09	0.06
	PNI	No	79	13	1			
		Yes	15	6	3.98	1.28	12.40	0.02
Local RFS	DOI	Continuum	94	15	1.06	0.91	1.24	0.45
	PORT	No	76	12	1			
		Yes	18	3	0.30	0.05	1.98	0.21
	PNI	No	79	10	1			
		Yes	15	5	4.03	1.09	14.8	0.04
OS	DOI	Continuum	94	9	1.07	0.90	1.28	0.45
	PORT	No	76	8	1			
		Yes	18	1	0.33	0.025	5.28	0.43

DFS = disease free survival, RFS = relapse free survival, OS = overall survival, DOI= depth of infiltration, PORT = postoperative radiotherapy, PNI = perineural invasion, HR = hazard ratio.

## Data Availability

The data presented in this study are available on request from the corresponding author. The data are not publicly available due to ethical reasons.
